# K_2_Fe(C_2_O_4_)_2_: An Oxalate Cathode for Li/Na-Ion Batteries Exhibiting a Combination of Multielectron Cation and Anion Redox

**DOI:** 10.1021/acs.chemmater.3c00063

**Published:** 2023-03-13

**Authors:** Atin Pramanik, Alexis G. Manche, Moulay Tahar Sougrati, Alan V. Chadwick, Philip Lightfoot, A. Robert Armstrong

**Affiliations:** † School of Chemistry, University of St. Andrews, Fife, St. Andrews KY16 9ST, United Kingdom; ‡ Université de Montpellier, 2 Place Eugène Bataillon - CC 1502, 34095 Montpellier Cedex 5, France; § ALISTORE-ERI, 80039 Amiens Cedex, France; ∥ School of Physical Sciences, University of Kent, Kent, Canterbury CT2 7NH, United Kingdom; ⊥ The Faraday Institution, Quad One, Harwell Science and Innovation Campus, Didcot OX11 0RA, United Kingdom

## Abstract

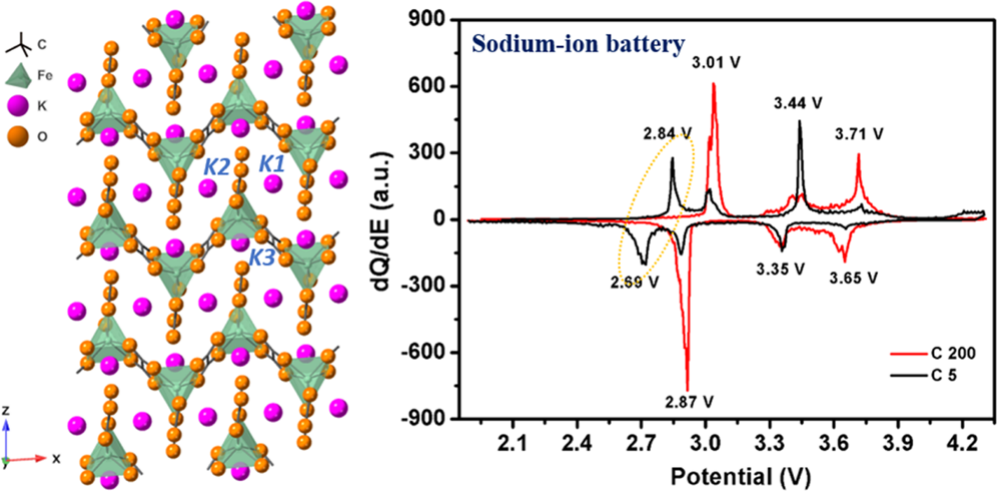

The development of multielectron redox-active cathode materials is a top priority for achieving high energy density with long cycle life in the next-generation secondary battery applications. Triggering anion redox activity is regarded as a promising strategy to enhance the energy density of polyanionic cathodes for Li/Na-ion batteries. Herein, K_2_Fe(C_2_O_4_)_2_ is shown to be a promising new cathode material that combines metal redox activity with oxalate anion (C_2_O_4_^2–^) redox. This compound reveals specific discharge capacities of 116 and 60 mAh g^–1^ for sodium-ion batterie (NIB) and lithium-ion batterie (LIB) cathode applications, respectively, at a rate of 10 mA g^–1^, with excellent cycling stability. The experimental results are complemented by density functional theory (DFT) calculations of the average atomic charges.

## 1 Introduction

There is an essential requirement for increased energy storage for a sustainable planet, and this represents a major driving force in the development of improved rechargeable batteries.^1^ In this respect, the search for promising new electrode materials for rechargeable batteries is one of the key challenges. Among the various energy storage systems, lithium-ion batteries (LIBs) represent the most promising energy storage mechanism in portable devices due to their high operating voltage, energy density, long life cycle, and affordable cost. Recently, due to concerns over the supply and increasing cost of Li, there is growing interest in more sustainable sodium-ion batteries (NIBs). Sodium has an analogous electrochemical behavior to lithium but is considerably more abundant. However, the storage capacity and cycling stability of NIBs are generally inferior to their lithium counterparts due to the larger ionic radius of Na^+^ and large volume change upon Na^+^ extraction or insertion.^2^ Therefore, desirable electrode materials for NIBs should adopt robust and flexible crystal structures that can accommodate large sodium ions. At the same time, cost-effective and environmental-friendly energy storage systems to meet the huge current demands drive the search for new polyanionic compounds.^3^ Various positive electrode materials have been explored for sodium-ion batteries, such as layered sodium transition-metal oxides Na*_x_*MO_2_ (M = Mn, Ni, Fe, Co), olivine NaMPO_4_ (M = Fe, Mn), and sodium superionic conductor (NASICON) Na*_x_*M_2_(PO_4_)_3_ (M = V, Ti).^3−6^ Olivine type (NaTMPO_4_) and NASICON type (Na*_x_*MM′(PO_4_)_3_ with M and M′ = Ti, V, Cr, Mn, Fe, Co, Ni, etc.)^7^ have been investigated and show promising cycling stability.^6,8^ In particular, polyanionic compounds can work at a high operating potential due to the strong inductive effect, as well as benefitting from robust and stable frameworks due to strong covalent bonds. In the search for suitable transition metals, iron is the most desirable redox center due to its low cost, low toxicity, and variable oxidation state. Recent studies have introduced a few important families of potential polyanionic cathode materials (e.g., phosphates and sulfates); some recently reported materials include NaFePO_4_,^9^ Na_2_FeP_2_O_7_,^10^ NaFePO_4_F,^11^ Na_4_Fe_3_(PO_4_)_2_(P_2_O_7_),^12^ NaFe_3_(HPO_3_)_2_,^13^ Na_2_Fe_2_(SO_4_)_3_,^14^ and NaFe(SO_4_)_2_.^15^

Although most of the reported polyanionic materials are based on phosphate and sulfate groups, oxalates have recently emerged as a promising new class. Oxalate anions are potentially tetradentate ligands, in which the four oxygen atoms are coordinated with cationic centers, but can also function as a mono-, bi-, or tri-dentate ligand, and form mono- or poly-nuclear metal complexes. The high versatility of the oxalate ligand is exemplified by at least 15 different known coordination modes concerning metal centers. Recent examples of oxalate cathode activity include Na_2_Fe_2_(C_2_O_4_)_3_·2H_2_O,^16^ Li_2_Fe(C_2_O_4_)_2,_^17^ Na_2_Fe(C_2_O_4_)_2_(HPO_4_),^19^ Na_2_Fe(C_2_O_4_)F_2_,^18^ Fe_2_(C_2_O_4_)_3_·4H_2_O,^19^ KLi_3_Fe(C_2_O_4_)_3,_^20^ Na_2_M_2_(C_2_O_4_)_3_·2H_2_O (M = Mn, Fe, Co),^22^ and K_4_Na_2_[Fe(C_2_O_4_)_2_]_3_·2H_2_O.^23^ Some of these examples exhibit natural drawbacks, either containing structural water or being fabricated in the charged state (Fe^3+^),^21^ whereas positive electrodes are typically prepared in the discharged state (Fe^2+^) and act as a lithium/sodium source for the negative electrode during battery application. From these previous reports, it is observed that transition metals in oxalates display redox potentials lower than those of sulfates but comparable to those of phosphates, which reflects the electronegativity of (SO_4_)^2–^ > (C_2_O_4_)^2–^ > (PO_4_)^3–^. This implies an attractive class of prospective polyanionic positive electrode materials.

Goodenough and co-workers have attributed redox at high states of charge to oxidation of the oxide anions to form peroxo-like moieties in Li-rich metal oxides (Li_2_RuO_3_); such a mechanism is termed “anion redox”.^21−24^ This anionic redox phenomenon has drawn much interest because the capacity may be significantly enhanced if both anionic and cationic redox reactions take place in the same electrode material. This phenomenon has been quite widely observed, for example, in Li-rich layered oxides, sulfides, and layered sodium transition-metal oxides but is much less common in polyanionic systems.^23,25−27^ Additionally, oxide cathodes are prone to evolve O_2_, CO_2_, or CO irreversibly upon oxidation at higher potentials.^24,28^ In this context, in lithium-ion batteries, the only reversible materials rely on platinum-group elements such as Ir and Ru, which overcome gas liberation.^29,30^ Consequently, developing sustainable polyanionic cathodes applying both anionic and cationic redox couples represents a strategy to meet the requirements in terms of capacity, energy density, and safety. We have recently demonstrated such activity in oxalate Li_2_Fe(C_2_O_4_)_2._^17^

Inspired by this consideration, here we report a further iron-based oxalate compound, K_2_Fe(C_2_O_4_)_2_,^31^ as a positive electrode for lithium-ion and sodium-ion batteries. The oxidation/reduction mechanism is suggested to involve multiple-phase intercalation/deintercalation of Li/Na, via both the Fe^2+^/Fe^3+^ cationic redox couple and an oxalate anionic redox contribution. Mössbauer spectroscopy, X-ray absorption near edge structure (XANES) data, and Raman spectroscopy analysis as a function of charge/discharge state suggested that electrochemical oxidation resulted in less than one electron transfer through the Fe^2+^/Fe^3+^ couple, with no oxidation to Fe^4+^. This strongly implies that the extra contribution comes from oxalate anion redox. By correlating the detailed electrochemical performance with structural and spectroscopic data, we establish that multielectron cation and anion redox is involved in this material. To compare the behavior of K_2_Fe(C_2_O_4_)_2_ with the previously reported Li_2_Fe(C_2_O_4_)_2_ material, and identify possible signatures for anion redox in oxalates, we have prepared Li_2_Fe(C_2_O_4_)_2_ and studied its performance vs Na^+^/Na.

## 2 Experimental Section

### 2.1 Synthesis and Characterization

#### 2.1.1 Synthesis of K_2_Fe(C_2_O_4_)_2_

Single crystals of the targeted compound were synthesized by a hydrothermal method. First, iron(II) chloride tetrahydrate, potassium carbonate, and oxalic acid were mixed homogeneously in the molar ratio of 1:3:3.8 in a mortar and pestle and immediately transferred to a 23 mL Teflon-lined autoclave. The autoclave was placed in an oven at 190 °C for 3 days and allowed to cool down to room temperature naturally. Subsequently, 2 mL of deionized H_2_O was added to the autoclave, which was then kept at 190 °C for 3 days and cooled in air. The contents of the autoclave were decanted onto filter paper, washed several times with methanol, and dried in an oven at 60 °C for 4 h. The resulting orange crystals were separated manually from the noncrystalline side products.

#### 2.1.2 Synthesis of Li_2_Fe(C_2_O_4_)_2_

Single crystals of the target compound were also synthesized hydrothermally. Iron(II) chloride hexahydrate (1.5 mmol), oxalic acid dihydrate (4 mmol), and lithium carbonate (3 mmol) were mixed homogeneously in a 23 mL Teflon-lined autoclave. The autoclave was placed in an oven at 190 °C for 6 days and cooled down to room temperature. The contents of the autoclave were filtered, washed several times with water and acetone, and dried in an oven at 60 °C for 4 h.

#### 2.1.3 Powder X-ray Diffraction (PXRD)

A powder X-ray diffraction (PXRD) pattern for the K_2_Fe(C_2_O_4_)_2_ sample was obtained on a Stoe STADI P diffractometer using Mo Kα_1_ radiation (λ = 0.70930 Å) with a position-sensitive Mythen linear detector. The data were recorded in the 2θ range of 3–45° at room temperature in capillary (0.5 mm diameter) Debye–Scherrer mode. The PXRD pattern of Li_2_Fe(C_2_O_4_)_2_ was recorded on a Stoe STADI P diffractometer operating in either transmission mode or Debye–Scherrer mode with Cu Kα_1_ radiation (λ = 1.5406 Å) in the 2θ range of 10–90°. Rietveld refinements were carried out using the GSAS software and Topas Academic V6.^32,33^ Morphology and mapping of the sample were recorded using a JEOL JSM-6700F scanning electron microscope (SEM), and the instrument was also equipped with a field emission gun (FEG) electron source. Secondary electron images were recorded with a tungsten filament electron source using an accelerating voltage of 5 kV for the hand-ground pristine sample, and 15 kV for ball-milled cathode samples. A retractable backscattered electron detector was applied for atomic number contrast imaging.

### 2.2 Electrochemistry

The crystalline material was first ball-milled for 30 min to make a fine powder using a Fritsch Pulverisette 8 mill. Then, 0.6 g of powdered active material was mixed with 0.3 g of Super C65 conductive carbon black using the same procedure for another 30 min. The composite powder was then ground with 0.1 g poly(tetrafluoroethylene) (PTFE) binder until homogeneous mixing was achieved. CR2325 coin cells were assembled in an Ar-filled glovebox and used for evaluation of electrochemical performance. The cells consisted of a disc electrode containing 10–12 mg active material, sodium metal as a counter/reference electrode, a glass fiber separator (Whatman, GF/F) and the electrolyte 1 M NaClO_4_ in propylene carbonate containing 3% fluoroethylene carbonate by weight for NIB. In the case of LIB, Li metal was employed as the counter electrode, with LP30 (1 M LiPF_6_ in ethylene carbonate: dimethyl carbonate = 1:1) as the electrolyte. The half-cells were tested by galvanostatic cycling in the potential window of 1.9–4.3 V for NIB and 1.9–4.5 V for LIB using a Biologic Macpile II system. To prepare samples for ex situ measurements, binder-free pellet electrodes were used. For all ex situ measurements, cycled cells were transferred to an Ar-filled glovebox bef ore opening and the active material was extracted. The electrodes were rinsed carefully with dry dimethyl carbonate to remove residual electrolyte and then left under vacuum for 12 h to ensure all of the solvent had evaporated.

### 2.3 Mössbauer Spectroscopy

Mössbauer spectra were recorded on absorbers prepared under argon (coffee bags) at room temperature. Each absorber contains 30–40 mg cm^–2^ active material recovered by washing with dimethyl carbonate (DMC). The spectrometer operates in the constant acceleration transmission geometry. The γ-ray source (^57^Co/Rd, 850 MBq) is maintained at room temperature. The isomer shift scale is calibrated using pure α-Fe standard. The obtained data were fitted using a least-squares method and a combination of Lorentzian lines with the MOSFIT program.

### 2.4 X-ray Absorption Spectroscopy (XAS)

The iron K-edge X-ray absorption spectra were recorded at the Diamond Light Source at the B18 beamline (Oxfordshire, United Kingdom). The 10 mg powder samples were ground with 150 mg of cellulose for ∼30 min and pressed into 13 mm diameter pellets inside the Ar-filled glovebox. The pellets were placed in the sample holder and sealed into aluminium bags. All spectra were recorded three times. The recorded XAS spectra were aligned, merged, and normalized using Athena and Artemis software.^34^

### 2.5 Raman Spectroscopy

Raman spectra were recorded using a Renishaw inVia Qontor confocal Raman microscope with a 532 nm laser and 1800 l mm^–1^ grating from 100 to 1900 cm^–1^. To measure the ex situ Raman spectra, the K_2_Fe(C_2_O_4_)_2_ pellet electrodes were assembled with the sample, Super C65 conductive carbon, graphite, and PTFE with a weight ratio of 65:15:10:10, respectively. The electrodes stopped after 15 cycles at the end of charge, and the end of discharge was sealed in the Ar-filled glovebox in an optical cell (EL-Cell) prior to Raman experiments to avoid air exposure.

### 2.6 Computational Methods

Spin-polarized calculations were performed using density functional theory (DFT), as implemented in the Vienna ab initio simulation package (VASP). This package is based on the plane-wave basis, and the projector augmented wave (PAW) representation. The Perdew–Burke–Ernzerhof (PBE) functional within the generalized gradient approximation was used to find optimized structures of K*_x_*Fe(C_2_O_4_)_2_ with K^+^ sites partially and fully filled. DFT calculations using standard functionals cannot accurately capture the properties of strongly correlated Fe d orbitals due to the self-interaction error inherent to these functionals. This limitation is often encountered in materials that have strongly localized orbitals, such as transition-metal d states. To overcome this challenge, the +U Hubbard correction has been applied to generalized gradient approximation (GGA) functionals for many materials. In our calculations, we have applied a U value of 4.0 eV to address the interactions of Fe d-electrons and improve the accuracy of the results. This value was chosen based on previous research on oxalate-based materials with Fe as a transition metal.^16,18,35,36^ A kinetic energy cutoff of 520 eV was employed. Geometry optimizations of the unit cell were made by relaxing the atomic coordinates with forces less than 0.01 eV Å^–1^ and energy change smaller than 10^–5^ eV. A Γ-centered 3 × 4 × 2 *k*-point mesh was used for geometry optimizations and total energy calculations, and then, a denser, 6 × 8 × 4, *k*-point mesh was used in calculations to obtain electronic densities of states. The charge/discharge process was simulated by removing K-atoms one by one from K*_n_*Fe_4_(C_2_O_4_)_8_, with *n* varying from 8 to 3 (see Computational Methods to find the most stable configuration, Figure S10 and Table S4 in the Supporting Information). The atomic charges were analyzed using the Bader method and based on the algorithm developed by Henkelman et al.^37^

## 3 Results and Discussion

**Figure 1:**
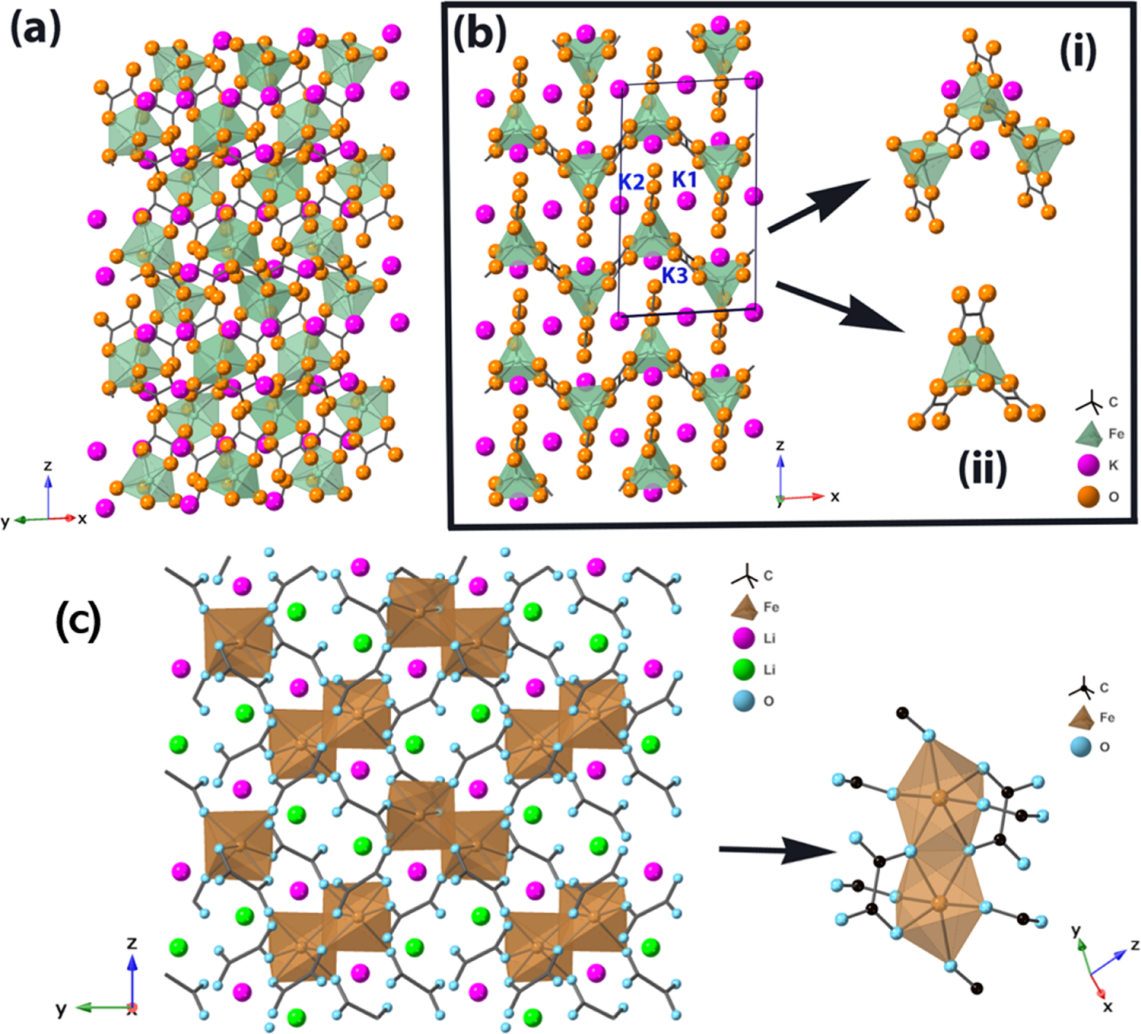
(a) Extended crystal structure of K_2_Fe(C_2_O_4_)_2_ viewed along the (110) plane to show its Fe^II^O_6_ motifs. (b) Interdigitation of the [Fe(C_2_O_4_)_2_]*_n_*^2*n*–^ chains showing in the packing diagram along the *b*-axis, and the corresponding positions of the three distinct potassium ions. (c) Three-dimensional crystal structure of Li_2_Fe(C_2_O_4_)_2_, where two FeO_6_ octahedra form a [Fe_2_O_10_] dimer connected by planar oxalate groups.

As reported previously, K_2_Fe(C_2_O_4_)_2_ has a monoclinic crystal structure with space group *P*2/*c*.^38^ The extended crystal structure is shown in Figure 1a. Figure 1b displays the crystal packing along the *b*-axis and the interdigitation of the infinite zigzag [Fe(C_2_O_4_)_2_]*_n_*^2*n*–^ chains separated from each other by potassium ions. In this structure, each Fe^II^ metal center is coordinated by three oxalate ligands in the usual chelating mode (Figure 1b(ii)). The Fe^II^ ion has a rare trigonal-prismatic coordination geometry. These anionic chains contain the two different types of oxalate ion, one of which is simply coordinated to a single Fe^II^ ion (Figure 1b(ii)) while the others bridge two metal ions in the common bis-chelating mode (Figure 1b(i)). There are three potassium environments with considerable dissimilarity in their coordination: K1 occupies a distorted square antiprism, K2 lies in an irregular nine-coordinate geometry, and K3 is six-coordinated in a distorted octahedral environment.

**Figure 2:**
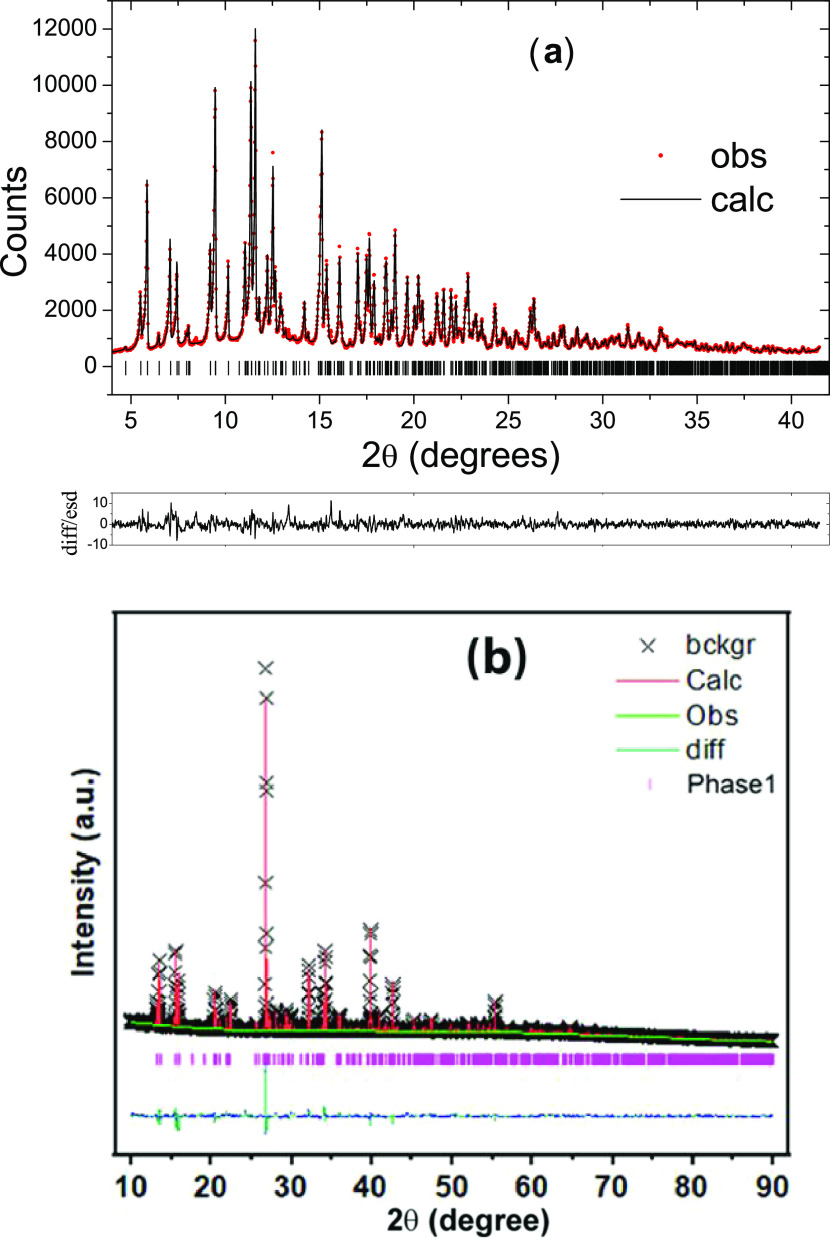
Rietveld refinement of hand-ground powder samples at room temperature. Lattice parameters at ambient temperature: (a) K_2_Fe(C_2_O_4_)_2_, lattice parameters *a* = 8.6007(7) Å, *b* = 6.9201(6) Å, *c* = 14.7286(11) Å, and β = 92.795(6)°, *wR*_p_ = 0.0465, *R*_p_ = 0.0347; and (b) Li_2_Fe(C_2_O_4_)_2,_ lattice parameters *a* = 7.4084(2) Å, *b* = 9.9882(2) Å, *c* = 9.2131(2) Å, and β = 110.909(1)°, *wR*_p_ = 0.0291, *R*_p_ = 0.0169, χ^2^ = 6.331.

The crystal structure of Li_2_Fe(C_2_O_4_)_2_ is shown in Figure 1c, viewed along the *a*-axis. Li_2_Fe(C_2_O_4_)_2_ is monoclinic with space group *P*2_1_/*n* (CCDC no. 1416422).^17^ A small Fe unit of the crystal structure is shown that reveals FeO_6_ octahedra motifs (Figure 1c, right), which share their edges to form [Fe_2_O_10_] dimers. The Fe_2_O_10_ dimers are connected through the oxalate groups to form a three-dimensional framework [Fe(C_2_O_4_)_2_]^2–^, with lithium ions situated in the interstitial sites. There are two lithium sites (Li1, green and Li2, magenta) with coordination numbers 4 and 5, respectively. Rietveld refinement confirmed the phase purity of the samples K_2_Fe(C_2_O_4_)_2_ (Figure 2a) and Li_2_Fe(C_2_O_4_)_2_ (Figure 2b).

The as-prepared pristine materials were ball-milled for half an hour to reduce the particle size. To increase the electronic conductivity, carbon Super C65 was added with further ball-milling, as outlined in the Experimental Section. The morphology of the pristine sample showed a particle size of ∼1.5 to 2 μm (Figure S1), but after ball-milling it was reduced to ∼0.6 μm (Figure S2a,b). The SEM image of the composite material is shown in Figure S2c, which reveals the homogeneous distribution of conductive carbon C65. Energy-dispersive X-ray analysis (EDX) and corresponding elemental mapping were performed (Figure S3a,b), supporting a homogeneous distribution of all elements throughout the sample. SEM images for Li_2_Fe(C_2_O_4_)_2_ showing pristine, ball-milled, and composite materials with C65 carbon were recorded (Figure S4).

### 3.1 Electrochemical Characterization in Sodium-Ion Battery (NIB)

**Figure 3:**
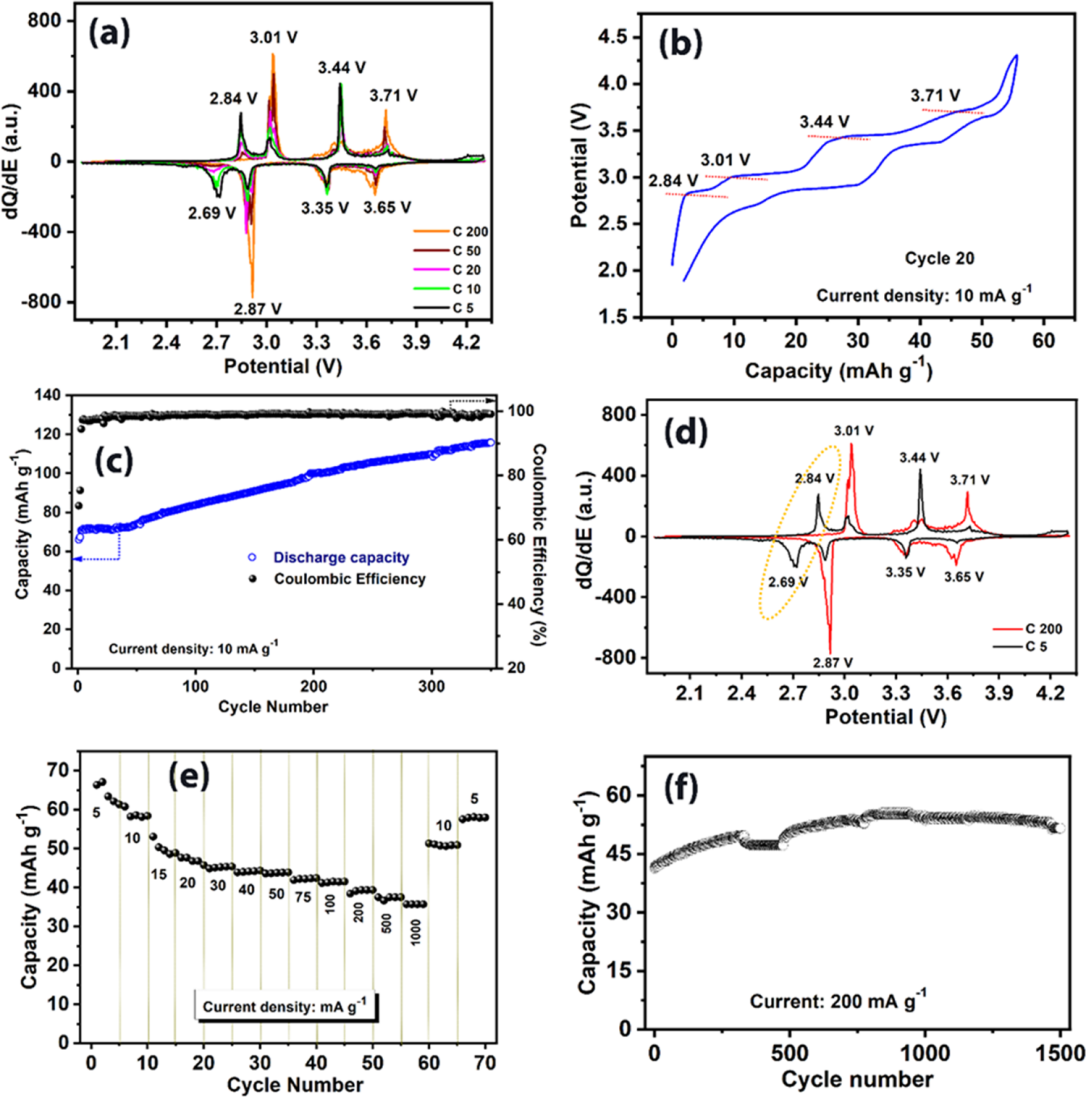
Half-cell performance of K_2_Fe(C_2_O_4_)_2_ as a NIB cathode. (a) Differential capacity plots at various cycle intervals for the first 200 cycles. (b) Galvanostatic voltage profile at the 20th cycle. (c) Cycling performance concerning cycle number and corresponding Coulombic efficiency at a current density of 10 mA g^–1^. (d) Differential capacity plots for the 5th and 200th cycles. (e) Rate capability at various current densities (5–1000 mA g^–1^). (f) High-rate (200 mA g^–1^) cycling performance.

The electrochemical properties of K_2_Fe(C_2_O_4_)_2_ were evaluated in coin cells. Differential capacity plots for a range of cycle numbers cycled between 1.9 and 4.3 V are shown in Figure 3a. The initial sodiation/desodiation reveals four pairs of oxidation and reduction processes at 2.84/2.69, 3.01/2.87, 3.44/3.35, and 3.71/3.65 V, respectively. Figure 3a reveals that there is no significant change in anodic and cathodic peak positions from cycle 5 to 20. Figure 3b shows the charge–discharge profile after 20 cycles, revealing the corresponding four plateaus. Figure 3c shows the continuous cycling performance of the electrode material up to 350 cycles. The initial discharge capacity of the material was 66 mAh g^–1^ at a rate of 10 mA g^–1^. A progressive increase in the capacity is observed, reaching 116 mAh g^–1^ after 350 cycles with Coulombic efficiency approaching 100%. The theoretical capacity of K_2_Fe(C_2_O_4_)_2_ is 87 mAh g^–1^ assuming complete oxidation of Fe^2+^ to Fe^3+^. The increase in capacity on extended cycling can be attributed to an electrochemical grinding effect that reduces diffusion lengths and improves electronic conductivity. A similar phenomenon has been observed for lithium-rich iron sulfide^23^ and Li_2_Fe(C_2_O_4_)_2_ when used as a LIB cathode material.^17^ However, there is clearly an additional capacity contribution beyond that obtained from oxidation from Fe^2+^ to Fe^3+^, which is discussed below.

Figure 3d shows the differential galvanostatic profiles for cycle numbers 5 and 200. The highlighted region in the plot shows that after 200 cycles, there are three pairs of peaks instead of the four seen in cycle 5. The 2.84/2.69 V process is progressively lost on extended cycling. This evolution in electrochemical behavior strongly implies that the progressive exchange of potassium for sodium occurs on extended cycling. The electrochemical performance of K_2_Fe(C_2_O_4_)_2_ was further characterized by an investigation of a range of different current densities (Figure 3e). From an initial discharge capacity of 66 mAh g^–1^ at a rate of 5 mA g^–1^, the capacity remained at 43 mAh g^–1^ (∼65.2% retention of the initial capacity) for a 20-fold increase in current density (100 mA g^–1^). A further increase in cycling rate to 500 mA g^–1^ and 1 A g^–1^ had little effect on the discharge capacity, which remained at 38 and 36 mAh g^–1^, respectively (57.6 and 54.5% retention of the initial capacity). Once the rate was restored to the initial current density (5 mA g^–1^), the discharge capacity reached 63 mAh g^–1^ (∼95.5%). Such rate performance compares favorably to recently reported polyanionic materials in the literature.^8^ Additionally, a cell was cycled at 200 mA g^–1^ for 1500 cycles to investigate the long-term cycling stability of K_2_Fe(C_2_O_4_)_2_ at a high current density (Figure 3f). Initially, the reversible capacity was 41 mAh g^–1^; after a few cycles, it progressively increased and stabilized at 53 mAh g^–1^. The excellent rate performance and long-term cycling stability demonstrate the potential of K_2_Fe(C_2_O_4_)_2_ as a NIB cathode.

### 3.2 Electrochemical Characterization in Lithium-Ion Battery (LIB)

**Figure 4:**
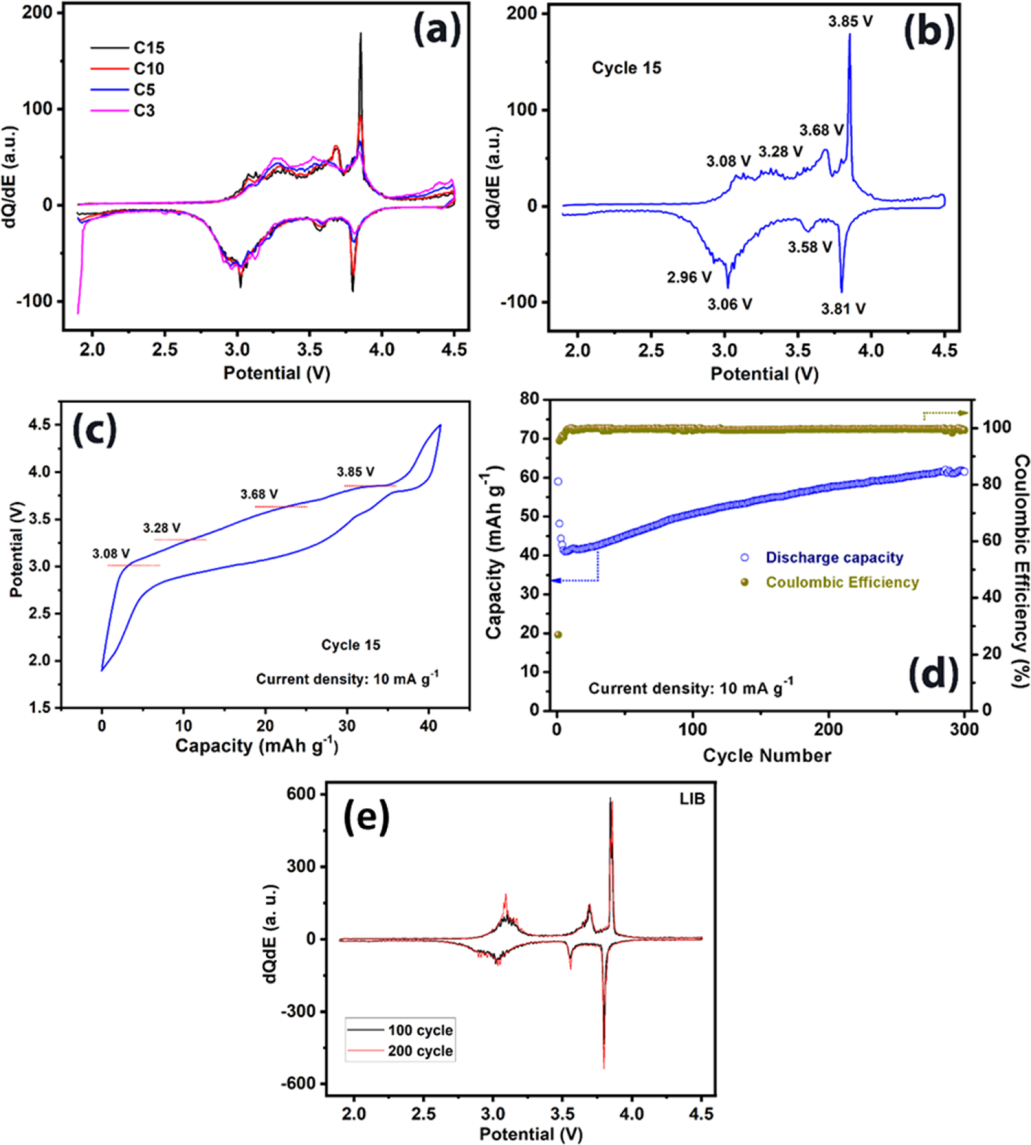
Half-cell performance of K_2_Fe(C_2_O_4_)_2_ as a LIB cathode. (a) Differential capacity plots for 3rd, 5th, 10th, and 15th cycles. (b) Differential capacity plots of the 15th cycle with four oxidation/reduction peaks assigned. (c) Galvanostatic voltage profile at the 15th cycle. (d) Cycling performance and corresponding Coulombic efficiency at a current density of 10 mA g^–1^. (e) Differential capacity plots for the 100th and 200th cycles.

The electrochemical performance of K_2_Fe(C_2_O_4_)_2_ as a positive electrode for LIBs was evaluated by galvanostatic cycling in a half-cell configuration. Figure 4a shows differential capacity plots for a range of cycles from 3 to 15 at a rate of 10 mA g^–1^ in the voltage window 1.9–4.5 V. Figure 4b displays the d*Q*/d*E* vs potential plot for cycle 15, indicating four oxidation/reduction processes at 3.08/2.96, 3.28/3.06, 3.68/3.58, and 3.85/3.81 V, respectively. Figure 4c displays the galvanostatic voltage profile at the 15th cycle at 10 mA g^–1^, which reveals the corresponding four oxidation/reduction plateaus. The d*Q*/d*E* vs potential plots for high cycle number (100 and 200 cycles, Figure 4e) shows three main processes, which are analogous to the behavior observed when cycled vs sodium. As discussed below, there are also strong similarities to the behavior of Li_2_Fe(C_2_O_4_)_2_.^17^Figure 4d displays the cycling performance for 300 cycles at a rate of 10 mA g^–1^. After an initial discharge capacity of 60 mAh g^–1^, the capacity drops for the first few cycles before stabilizing and gradually increasing to reach a value of 62 mAh g^–1^ (∼103% of the capacity of the initial cycle) after 300 cycles. A similar increase in capacity with the cycle number to that found in NIB was observed for LIB, attributable due to the electrochemical grinding effect.

### 3.3 Comparison with Li_2_Fe(C_2_O_4_)_2_ in Sodium-Ion Battery (NIB)

**Figure 5:**
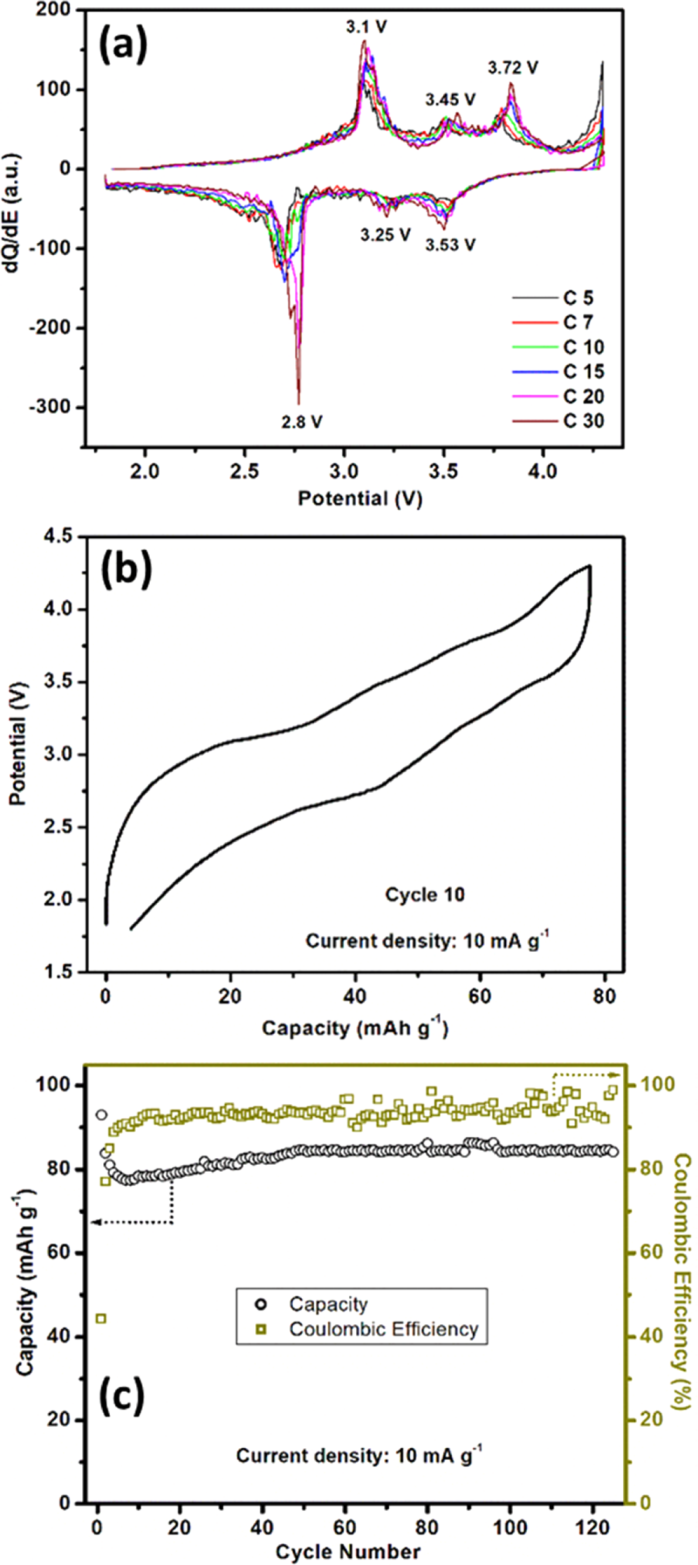
Half-cell performance of Li_2_Fe(C_2_O_4_)_2_ as a NIB cathode. (a) Differential capacity plots at various cycles for the first 30 cycles. (b) Galvanostatic voltage profile at the 10th cycle. (c) Cycling performance and corresponding Coulombic efficiency at a current density of 10 mA g^–1^.

To gain further insight into the electrochemical properties of K_2_Fe(C_2_O_4_)_2_ and identify possible signatures associated with oxalate redox, we investigated the electrochemical performance of Li_2_Fe(C_2_O_4_)_2_ as a positive electrode for sodium-ion batteries. We have previously reported the behavior of Li_2_Fe(C_2_O_4_)_2_ in LIBs including oxalate redox activity.^17^ Half-cells were cycled between 1.8 and 4.3 V. The initial sodiation/desodiation process exhibits three pairs of oxidation and reduction processes at 3.1/2.8, 3.45/3.25, and 3.72/3.53 V, respectively. Figure 5a reveals that there is no significant change in anodic and cathodic peak positions from cycles 5 to 30. Figure 5b shows the charge–discharge profile after 10 cycles exhibiting three plateaus corresponding to the three pairs of peaks observed in the d*Q*/d*V* plots. Figure 5c shows the cycling performance of the electrode material up to 125 cycles over the potential window 1.8–4.3 V at a cycling rate of 10 mA g^–1^.

The first cycle discharge capacity was 93.5 mAh g^–1^ (∼86% of the theoretical capacity). After an initial drop in capacity over the first few cycles, there was a subsequent increase prior to stabilization. Accompanying the capacity increase, the cell polarization decreases consistently with progressive activation of the electrode, which can be clearly shown by the potential vs time plot for the first 40 cycles (Figure S5, Supporting Information). At the end of 125 cycles, a discharge capacity of 85 mAh g^–1^ (∼91% of its initial capacity) was observed with a Coulombic efficiency of ∼96%. Comparison of the differential capacity plots for the 100th cycle for K_2_Fe(C_2_O_4_)_2_ and Li_2_Fe(C_2_O_4_)_2_, reveals essentially identical redox processes, albeit with higher polarization for Li_2_Fe(C_2_O_4_)_2_ (Figure S6, Supporting Information), hinting at a possible electrochemical signature associated with oxalate redox. This characteristic behavior should assist in the identification of further compounds exhibiting these properties. Work is underway to identify the precise origin of these processes, which will form the basis of a future publication.

### 3.4 Charge Compensation Mechanism

**Figure 6:**
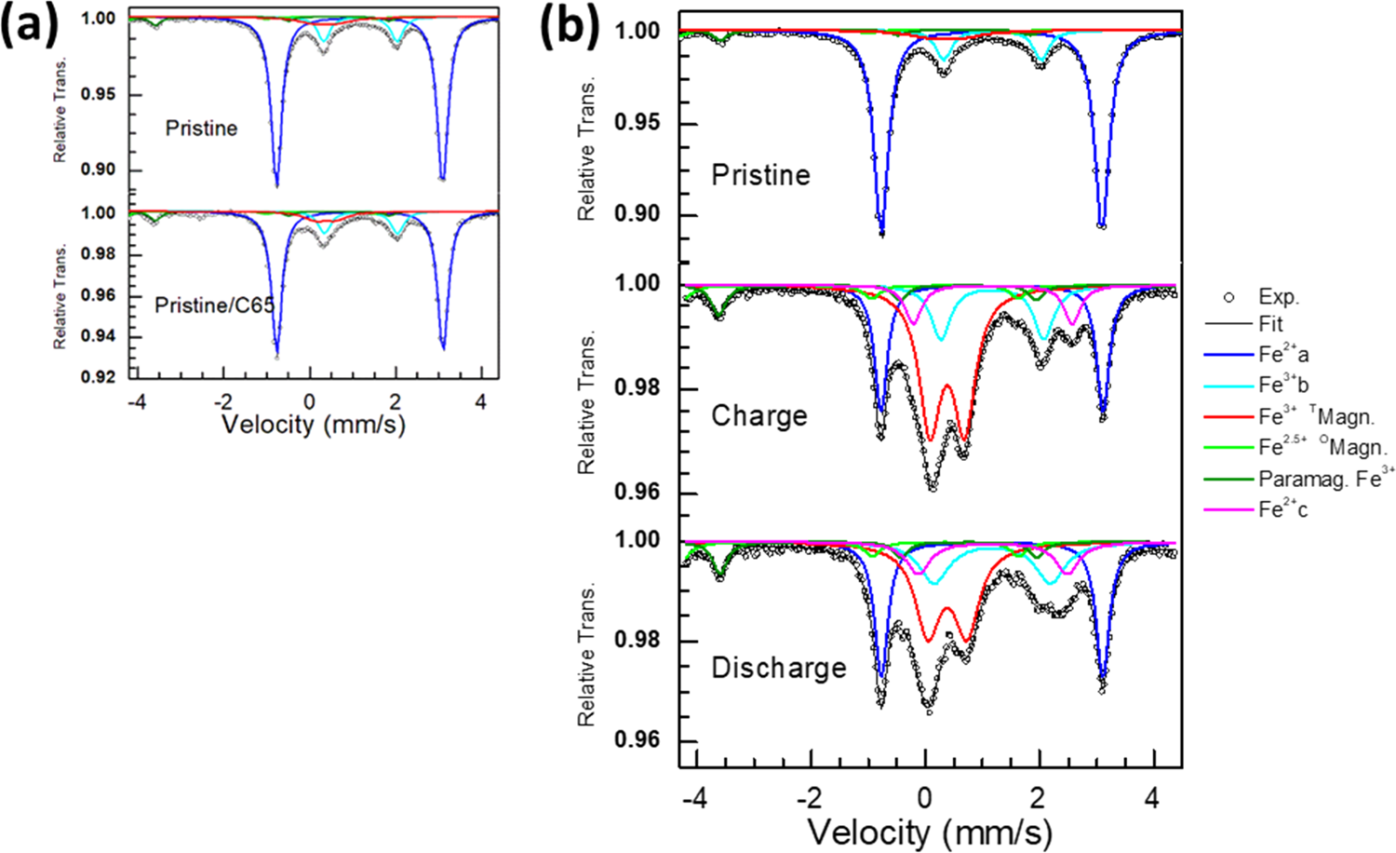
Room-temperature Mössbauer spectrum of the (a) pristine sample and conductive C65 carbon composite (pristine/C65), and (b) sample obtained from the full charge (4.3 V)/discharge (1.7 V) state of ex situ electrodes.

Mössbauer spectroscopy measurements were carried out for the pristine K_2_Fe(C_2_O_4_) and the composite with Super C65 carbon (Figure 6a). The main signal is 75% high-spin Fe^2+^ in octahedral sites with an isomer shift (δ) higher than 1 mm s^–1^. The main signal is divided into two parts with a high quadrupole splitting (Δ) of 3.86 mm s^–1^, and lower quadrupole splitting (Δ) of 1.71 mm s^–1^, suggesting high-spin Fe^2+^ in octahedral coordination. A slight magnetic contribution is also present with one sextet for tetrahedral Fe^3+^ (light green) and one sextet for octahedral Fe^2.5+^ (olive green). A trace amount of paramagnetic Fe^3+^ (red) is also present in the sample. There was no significant difference between the pristine and ball-milled powder composite with conductive carbon C65. The cationic Fe^2+^/Fe^3+^ redox process of the K_2_Fe(C_2_O_4_)_2_ sample was investigated by ex situ ^57^Co Mössbauer spectroscopy analysis for different charge/discharge stages over different potential windows (Figure 6b). The detailed fitted data are shown in the Supplementary Information (Table S1). Spectra were taken from samples after 10 charge/discharge and half-charge/discharge cycles for both sodium and lithium cells. Mössbauer spectra confirm some iron oxidation and reduction in the charge/discharge process. The spectra were taken from the 10th charge vs Na^+^/Na (4.3 V) and fitted with isomer shift (IS), the quadrupole splitting (QS), the line width (LW), and the absorption (Abs) parameters (Figure 6b). The blue and pink line in the fitted plot shows that there is an overall ∼38% change of Fe^3+^. At the 10th discharge vs Na^+^/Na (1.7 V), Fe^3+^ is reduced to Fe^2+^. The detailed fitted values are shown in the Supporting Information (Table S2). The green and red lines show the signatures of Fe^2+^/Fe^3+^ in both charged and discharged states after cycling. We also examined the Mössbauer spectra at half-charge/discharge (3.1 V) (Figure S7, Supporting Information). These data show intermediate behavior. Mössbauer spectroscopy reveals no trace of a Fe^3+^/Fe^4+^ redox couple.

After cycling vs Li^+^/Li, Mössbauer spectra reveal a similar degree of iron oxidation and reduction in the charge/discharge process, as indicated in Figure S8, Supporting Information. Comparison of the spectrum (fitted with four doublets) obtained after the 10th charge (up to 4.5 V) with that of pristine K_2_Fe(C_2_O_4_)_2_ reveals the partial oxidation of Fe^2+^ to Fe^3+^. On the other hand, for the fully discharged sample (1.9 V), there is a partial reduction to Fe^2+^ from Fe^3+^. There is a small amount of unreacted material due to the large amount of powder needed for ex situ analysis (40 mg cm^–2^).

**Figure 7:**
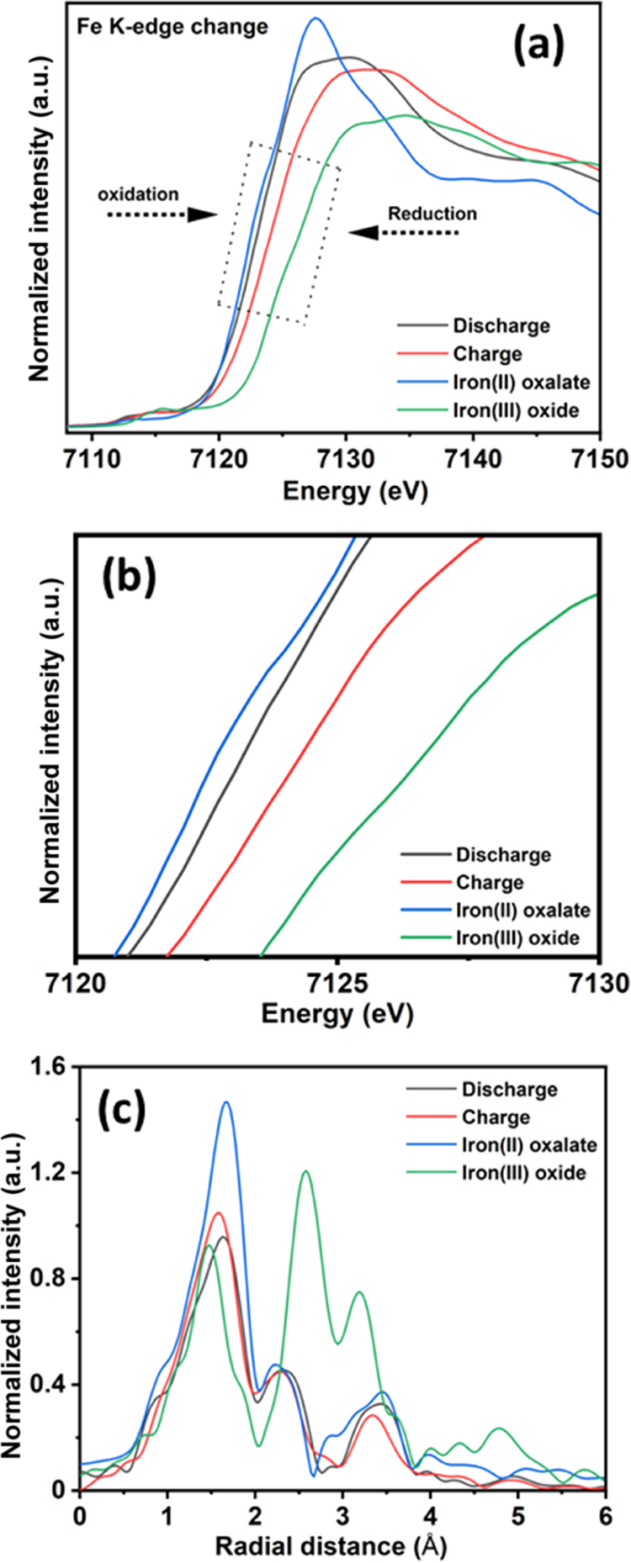
Validation of the charge/discharge mechanism and structural evolution of K_2_Fe(C_2_O_4_)_2_ at the 15th charge/discharge for NIB. (a) Normalized iron K-edge XANES data at the full charge/discharge state compared with commercial iron(II) oxalate and iron(III) oxide samples. (b) The magnified image of XANES spectra, highlighted in panel (a). (c) Iron K-edge EXAFS spectra and Fourier transform in the *R*-space for charge and discharge samples.

Additionally, the oxidation state evolution of K_2_Fe(C_2_O_4_)_2_ was characterized by X-ray absorption near-edge spectroscopy (XANES) measurements for 15th cycle charged/discharged samples for NIB (Figure 7). The spectra were collected for ex situ fully charged/discharged samples and commercial iron(II) oxalate and iron(III) oxide as references to compare the local Fe K-edge change (Figure 7a). The pre-edge peak at the energy range of 7111–7117 eV represents the symmetry of the Fe local structure and arises due to mixing of 3d and 4p orbitals.^17,39^ The changes in the coordination of the Fe atom can bring about changes in the intensity of pre-edge peaks. On the other hand, the characteristic shoulder at ∼7118 eV signifies an absence of FeO_4_ tetrahedra in the sample.^39^ The Fe K-edge XANES profile shifts toward higher energy during desodiation (charge), suggesting that Fe^2+^ ions are oxidized to Fe^3+^ (Figure 7b). Similarly, the Fe K-edge XANES profile shifts toward lower energy during sodiation (discharge), suggesting that Fe^3+^ ions are partially reduced to Fe^2+^. The Fe K-edge XANES profile at the end of the charge resembles iron(III) oxide but with a lower shift (Figure 7b), suggesting that the Fe^2+^ is not fully oxidized to Fe^3+^. The slight change of Fe K-edge during the charge/discharge process confirms that the FeO_6_ octahedral coordination is highly stable. These observations suggest that there is partial oxidation of Fe^2+^ to Fe^3+^, in agreement with the Mössbauer spectroscopy measurements (Figure 6). Similarly, on discharge, partial reduction of Fe^3+^ to Fe^2+^ is observed.

To determine the bond length and local structural environment around the Fe center, the extended X-ray absorption fine structure (EXAFS) spectra were recorded for charge/discharge samples, and the corresponding Fourier transform (FT) spectra are shown in Figure 7c. The *S*_0_^2^ value was determined from standard iron(II) oxalate and iron(III) oxide sample fitting and fixed to 0.730. During the charge process, the Fe–O bond length contracts because of the increasing oxidation state of iron. Similarly, the opposite trend was observed during the discharge process, with an increase in the Fe–O bond length observed. These observations imply the involvement of the Fe center in the charge/discharge process.

### 3.5 Experimental Evidence of Oxalate Anionic Redox Activity

**Figure 8:**
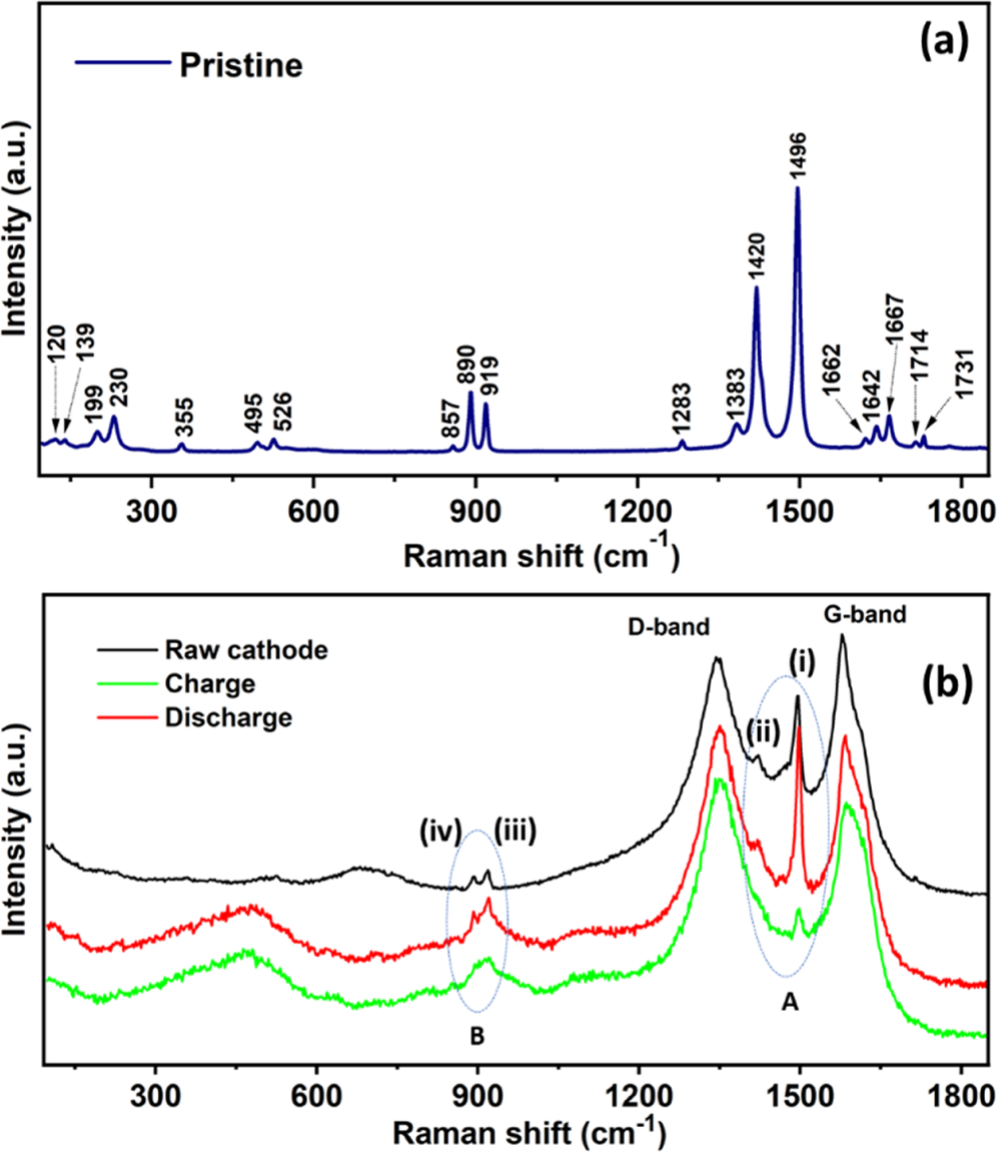
Characterization of oxalate anionic redox chemistry of K_2_Fe(C_2_O_4_)_2_ as a NIB cathode. (a) Raman spectroscopy analysis of the pristine sample, and (b) evolution of oxalate redox during charge and discharge state: raw cathode (black), charged cathode (green, in ex situ cell), and discharged (red, in ex situ cell) state cathode samples.

The above ex situ Mössbauer spectroscopy and XANES characterization of different states of charge/discharge samples confirm that there is a partial oxidation/reduction of iron 2+ to 3+ (Fe^2+^/Fe^3+^ redox) during the sodiation/desodiation process. However, this could not explain the capacity obtained from the charge/discharge process, suggesting that there could be some other electrochemical process involved. In this scenario, we recorded ex situ Raman spectra of samples at different states of charge. Raman spectroscopy is one of the most sensitive techniques to characterize the anion redox (oxalate redox) behavior. We measured ex situ Raman spectra after 15 cycles of charge/discharge. First, we collected Raman spectra for pristine samples (Figure 8a). Several vibrational peaks, two intense peaks at 890 and 919 cm^–1^, correspond to δ(O–C=O) bending and ν(C–C) stretching, respectively. Another two strong peaks at 1420 and 1496 cm^–1^ are the two main vibrations of the oxalate ligand corresponding to two stretching vibrations of (C=O).^17^ To determine the evolution of oxalate anion, we recorded Raman spectra of the raw electrode, together with the charged and discharged state of the NIB electrode (Figure 8b). Two strong and broad peaks at 1345 and 1579 cm^–1^ were observed due to the conductive carbon material, which represents D and G bands, respectively, which are typical for any carbon additives. The G band reflects the stretching motions between sp^2^-hybridized carbon atoms, whereas the D band originates due to the structural defects caused by the hybridization of carbon atoms. The central oxalate peak at (i) 1495 cm^–1^ originates from the C=O asymmetric stretching and is very sharp and prominent in the discharged state (red) with respect to the charged state (green). Similarly, another C=O stretching vibration (ii) 1420 cm^–1^ was also prominent in the discharged state, similar to the raw cathode (black), but was not observed on charge. In addition, (iv) symmetric C–C stretching at 890 cm^–1^ and (iii) bending O–C=O modes at 919 cm^–1^ were prominent at discharge, but a shoulder was observed in the charged state. The other vibration modes of the pristine sample are not observed for the cycled samples due to carbon additives, cell window, and noisy background, which is typical for ex situ samples. Comparing the spectra, the intensity evolution with respect to the state of charge confirms that C=O and O–C=O became more localized and sharper in the discharged state. Based on Raman spectroscopy characterization, the four prominent oxalate peaks support the implied oxalate anion redox during charge/discharge without CO_2_ evolution.

### 3.6 Computational Studies

**Figure 9:**
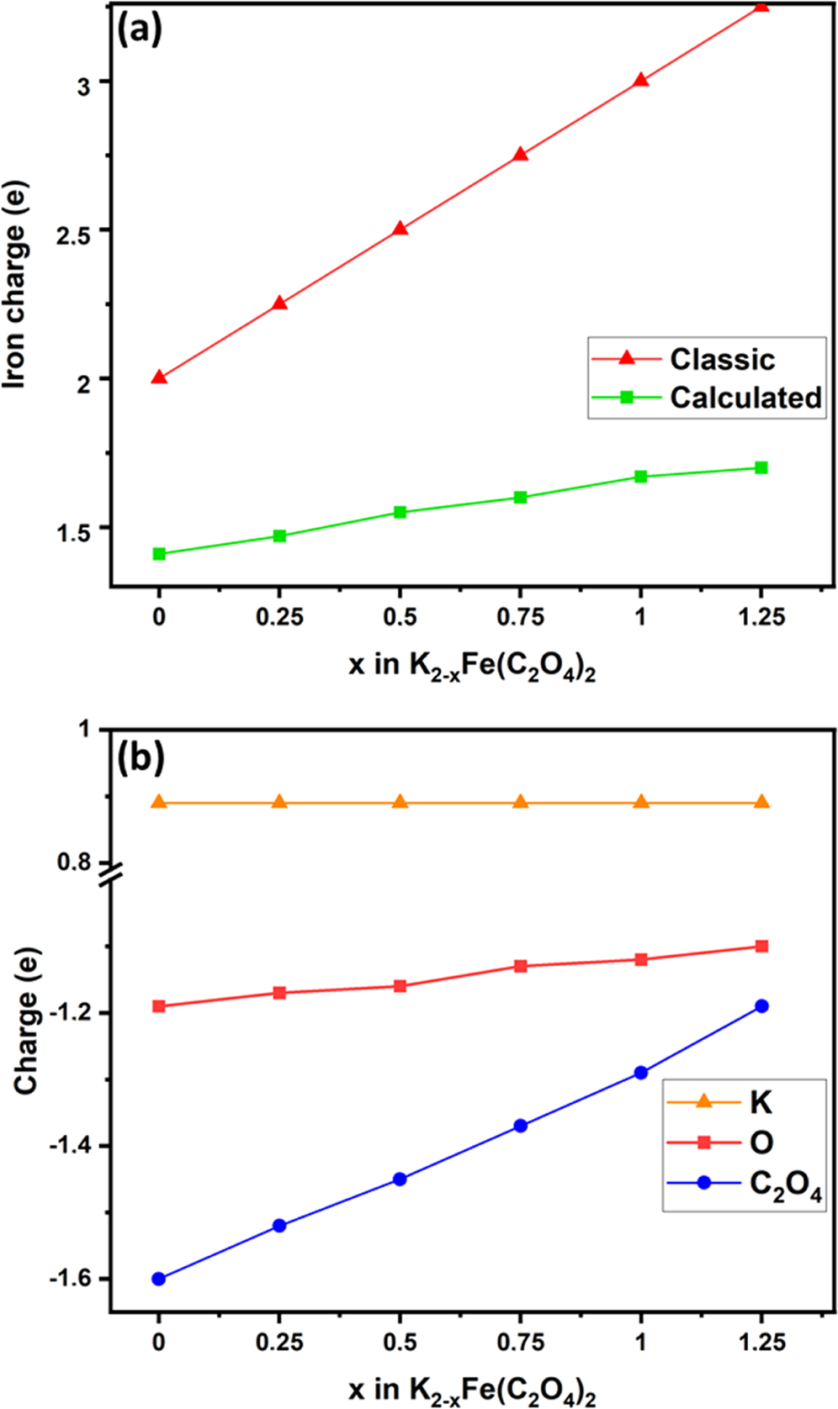
(a) Iron charges and (b) averaged charges for the K, O, and C_2_O_4_ group vs the K-content in K_2–*x*_Fe(C_2_O_4_)_2_.

As shown above, the experimental data suggest the presence of both cationic and anionic K_2_Fe(C_2_O_4_)_2_ redox processes. To probe this further, and to understand the redox activity during cycling, first-principles calculations were performed. The maximum capacity value measured in this study (∼116 mAh g^–1^) corresponds to approximately 5 atoms of K removed from the ideal K_8_Fe_4_(C_2_O_4_)_8_ unit cell used for calculations. In other words, more than half of the K-atoms may be extracted. From the classical valence bond theory (VBT) and when the K is completely incorporated in the structure, the charges for K, Fe, and C_2_O_4_ must be, respectively, δ(K) = +1, δ(Fe) = +2, and δ(C_2_O_4_) = −2. During charge and discharge, this balance must obviously be broken to allow the K ions to be removed. To maintain the charge balance, usually the oxidation only occurs on the transition metal, but here if all Fe^2+^ are oxidized to Fe^3+^, this gives a theoretical capacity of only ∼87 mAh g^–1^, which is lower than the maximum capacity measured experimentally (∼116 mAh g^–1^). It is for this reason that Bader charges have been calculated to better understand and verify the balance of charges on cycling. The calculated charge of iron δ(Fe) is shown in Figure 9a in green and deviates significantly from the linear relationship in red, indicating that the electrons lost during the extraction of the K ions cannot be fully compensated by iron oxidation. In Figure 9b, it is notable that the charge on the oxalate δ(C_2_O_4_) varies significantly while the charge on K remains the same (δ(K) = 0.89). This therefore highlights the polyanionic redox process in this material.

All of the compounds from K_8_Fe_4_(C_2_O_4_)_8_ to the hypothetical end compound K_3_Fe_4_(C_2_O_4_)_8_ were stable after structural optimization. When the number of K-atoms drops below 3, the lattice tends to have major structural changes due to the transformation of oxalate into carbon dioxide molecules. The calculated lattice parameters for the initial compound are *a* = 8.82 Å, *b* = 7.04 Å, *c* = 15.05 Å, and β = 92.87° with space group *P*2/*c*, which is in good agreement with the experimental results. In addition, iron is stable in this compound as high-spin Fe^2+^ and with a calculated magnetic moment of 3.76 μB. However, in the end compound K_3_Fe_4_(C_2_O_4_)_8_, only three quarters of the iron are high-spin Fe^3+^ with a calculated magnetic moment of 4.25 μB and one quarter of the iron remains in the high-spin Fe^2+^ configuration (3.76 μB), which confirms that the oxidation of the iron alone is not sufficient to account for the extraction of the K from the structure.

**Figure 10:**
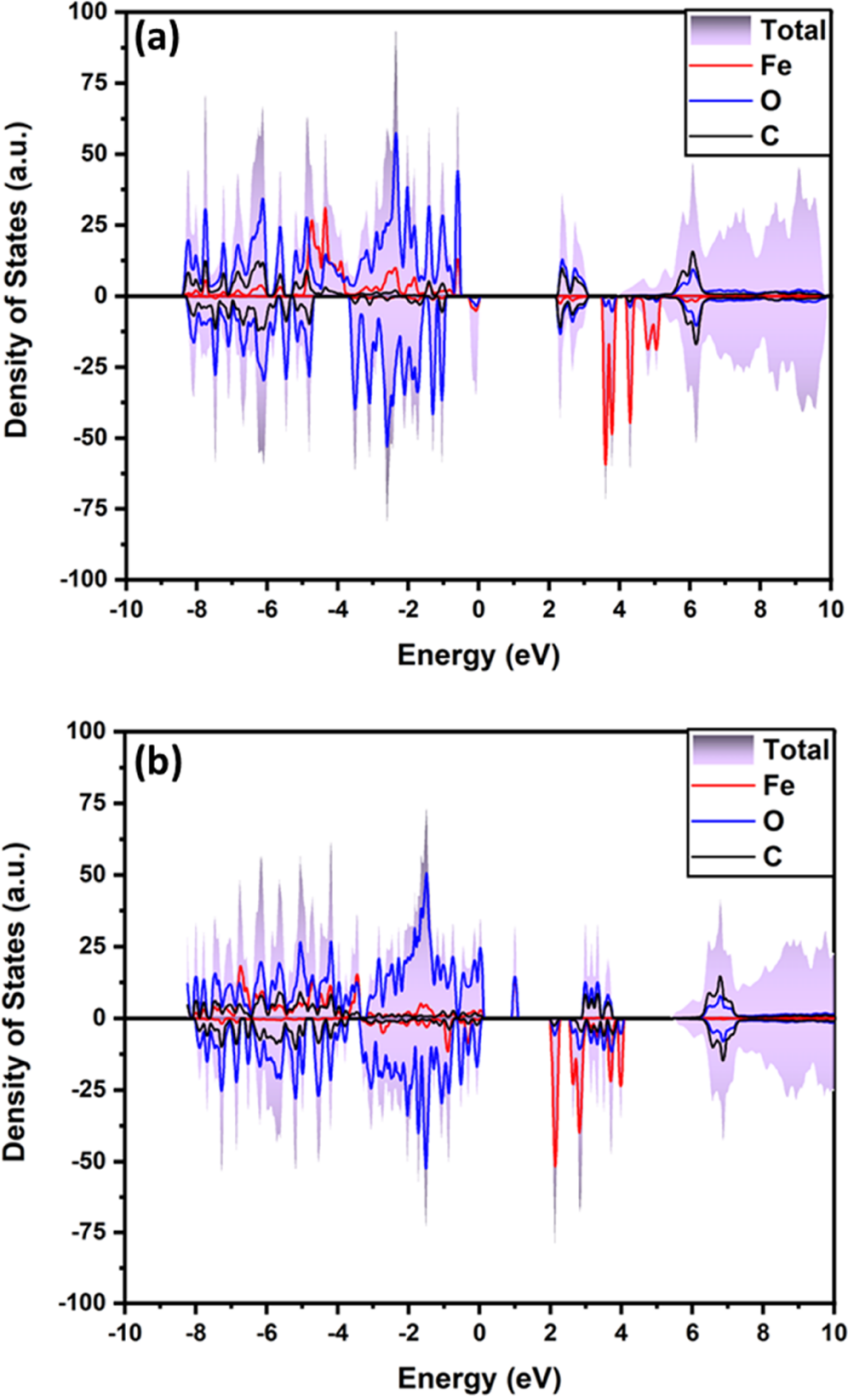
Density of states (DOS) of K_2–*x*_Fe(C_2_O_4_)_2_ for (a) *x* = 0 and (b) *x* = 1. The Fermi level is set at 0 eV.

Figure 10 displays the electronic density of states of K_2_Fe(C_2_O_4_)_2_ and KFe(C_2_O_4_)_2_ in the antiferromagnetic spin configuration with calculated band gaps of 2.26 and 2.07 eV, respectively. These band gaps are quite wide, and it is for this reason that the material requires significant carbon additives.

The nature of the electronic structure near the band edges provides information about the mechanism behind the insertion and extraction of K ions within the material. The figure shows that the top of the valence band below the Fermi level of K_2_Fe(C_2_O_4_)_2_ is mainly composed of highly localized Fe 3d states with a non-negligible overlap of O 2p states. When K^+^ ions start to be removed from the structure, the electrons from these two peaks are the first to be oxidized. This helps to explain the loss of electrons not only in Fe but also in O of oxalates during the initial redox process. After removal of half of the K^+^ ions (KFe(C_2_O_4_)_2_), the conduction band is now populated by Fe 3d states and a small overlap of O 2p states, showing that both Fe and O ions from the oxalate participate with electron loss from Fe are dominant, while anion redox is also evident.

## 4 Conclusions

In this work, K_2_Fe(C_2_O_4_)_2_, an iron-based low-cost transition-metal oxalate, was tested for the first time as a LIB/NIB cathode material. The compound delivers specific discharge capacities of 116 and 60 mAh g^–1^ at 10 mA g^–1^ current rate in NIB and LIB, respectively. The combination of both cationic (Fe^2+^/Fe^3+^) and oxalate anionic redox chemistry was explored during the charge/discharge process and characterized by ex situ Mössbauer spectroscopy, XANES, and Raman spectroscopy. The evolution of the oxalate group during charge/discharge was explored via ex situ Raman spectroscopy analysis, which implies that reversible anionic and cationic redox reactions occur simultaneously. The experimental observations were also validated by DFT studies.

## Supporting Information Available

SEM images of pristine and composites for K_2_Fe(C_2_O_4_)_2_ and Li_2_Fe(C_2_O_4_)_2_; detailed electrochemical characterization technique for Li_2_Fe(C_2_O_4_)_2_; potential vs time plot for Li_2_Fe(C_2_O_4_)_2_; differential capacity plot for both the samples at the 100th cycle; Mössbauer spectroscopy plots for different states of charge for LIB and NIB; and the corresponding fitting data tables (PDF)
