# Supplementary material for: K2Fe(C2O4)2: An Oxalate Cathode for Li/Na-Ion Batteries Exhibiting a Combination of Multielectron Cation and Anion Redox
Source: Chem Mater. 2023 Mar 13;35(6):2600–11. doi: 10.1021/acs.chemmater.3c00063 (PMC10061677; doi:10.1021/acs.chemmater.3c00063)
Supplement: Supplementary file 1 — cm3c00063_si_001.pdf [file cm3c00063_si_001.pdf]

Supporting Information

$K_2Fe(C_2O_4)_2$: An Oxalate Cathode for Li/Na-ion Batteries Exhibiting a Combination of Multielectron Cation and Anion Redox

Atin Pramanik†, Alexis G. Manche†◊, Moulay Tahar Sougrati§‡, Alan V. Chadwick‖‡, Philip Lightfoot†, A. Robert Armstrong*†‡◊*

†School of Chemistry, University of St Andrews, St Andrews, Fife KY16 9ST, United Kingdom

§Université de Montpellier, 2 Place Eugène Bataillon - CC 1502, 34095 Montpellier Cedex 5, France

‡ALISTORE-ERI, 80039, Amiens Cedex, France

‖ School of Physical Sciences, University of Kent, Canterbury, Kent, CT2 7NH, United Kingdom

◊ The Faraday Institution, Quad One, Harwell Science and Innovation Campus, Didcot, OX11 0RA, United Kingdom

* pl@st-andrews.ac.uk, ara@st-andrews.ac.uk

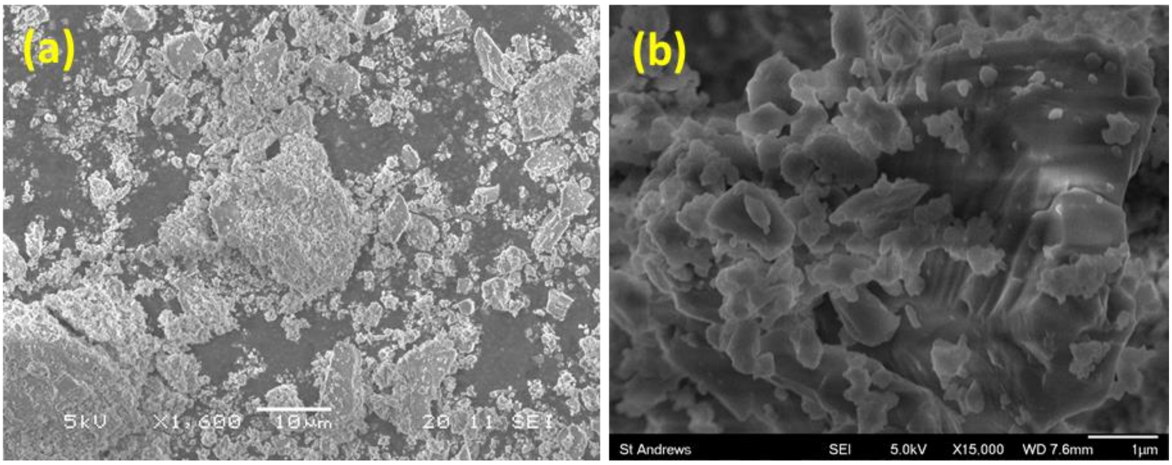

Figure S1. (a) Low and (b) high magnification SEM image of pristine $K_2Fe(C_2O_4)_2$.

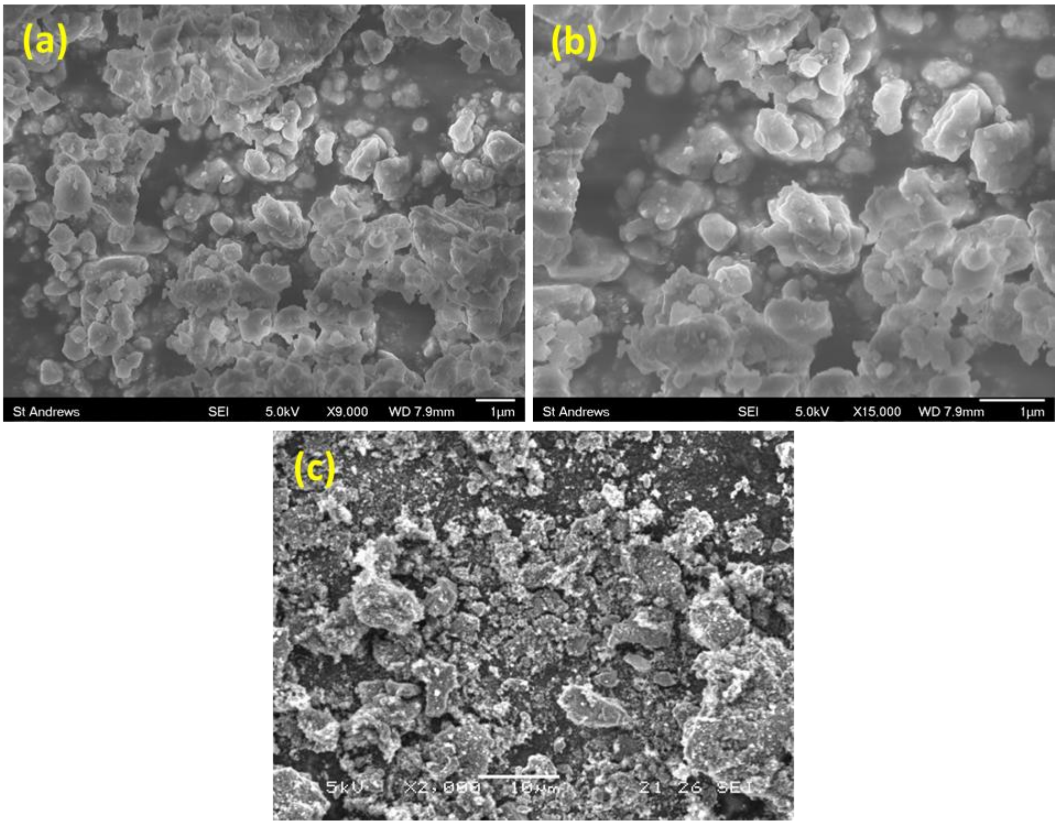

Figure S2. (a, b) Low and high magnification SEM image of pristine $K_2Fe(C_2O_4)_2$ after half an hour ball-milling. (c) SEM image of the ball-milled sample with C65 conductive carbon.

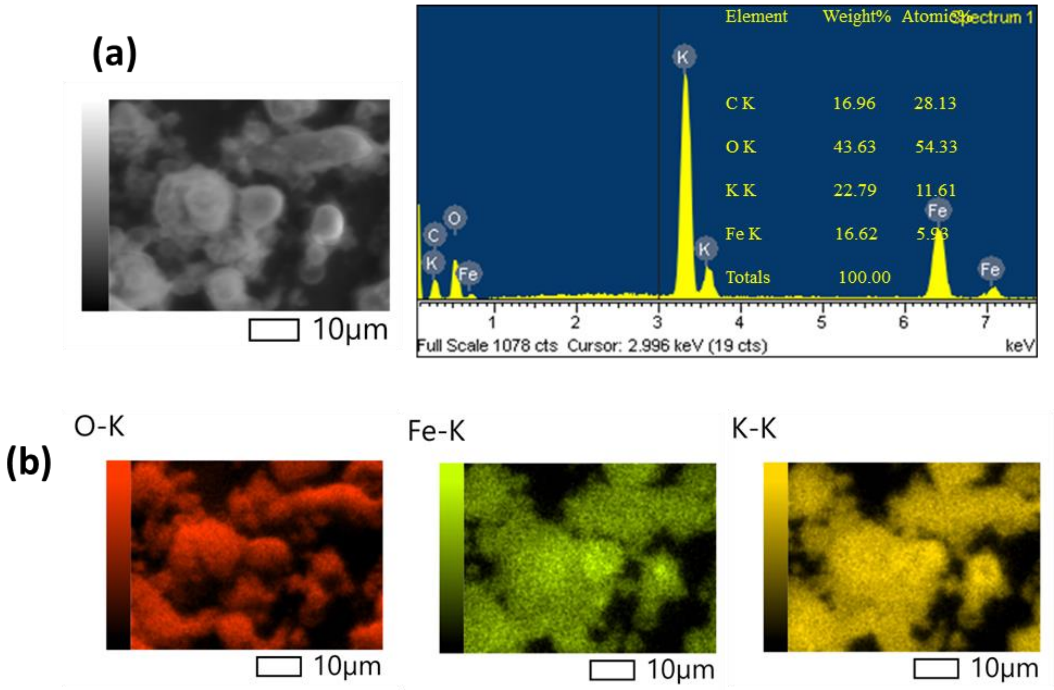

Figure S3. (a) SEM EDX elemental analysis of $K_2Fe(C_2O_4)_2$, and (b) corresponding elemental mapping.

Electrochemical characterization for $Li_2Fe(C_2O_4)_2$

The yellow crystalline material was the ball milled for 30 min to make a fine powder using a Fritsch Pulverisette 8 mill. 0.6 g of ball-milled powder was then mixed with 0.3 g Super C65 conductive carbon black and ball milled for another 30 min. After that, the composite powder was ground with 0.1 g polytetrafluoroethylene (PTFE) binder until homogeneous mixing was achieved. Typically, in the half-cell assembly, 6-7 mg active material was tested using CR2325 (NRC Canada) coin cells with Na metal as the anode, 1 M $NaClO_4$ in propylene carbonate with 3% fluoroethylene carbonate as the electrolyte for NIB. The half-cells were tested by galvanostatic charge-discharge in the potential window of 1.8-4.3 V for NIB at 10 mA g^{-1} current density using a Biologic Macpile II system.

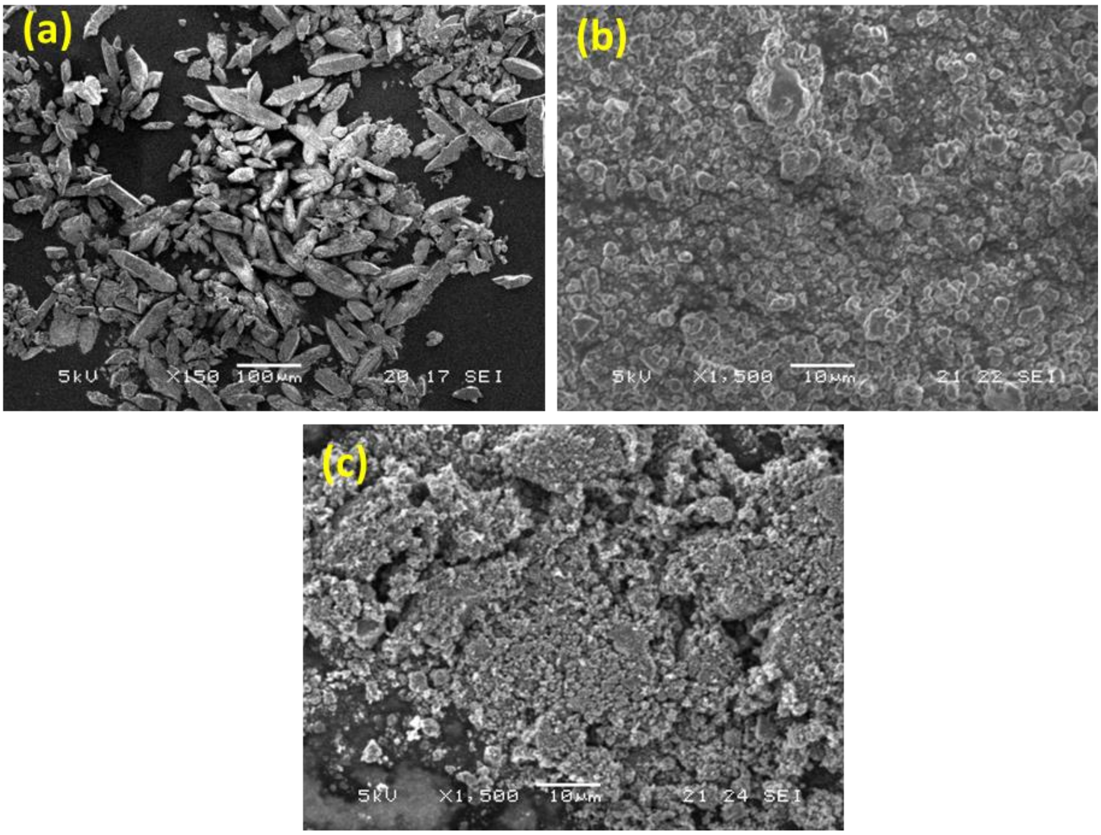

Figure S4. SEM images of $Li_2Fe(C_2O_4)_2$ (a) pristine, (b) ball-milled, and (c) sample with carbon C65 composite powder.

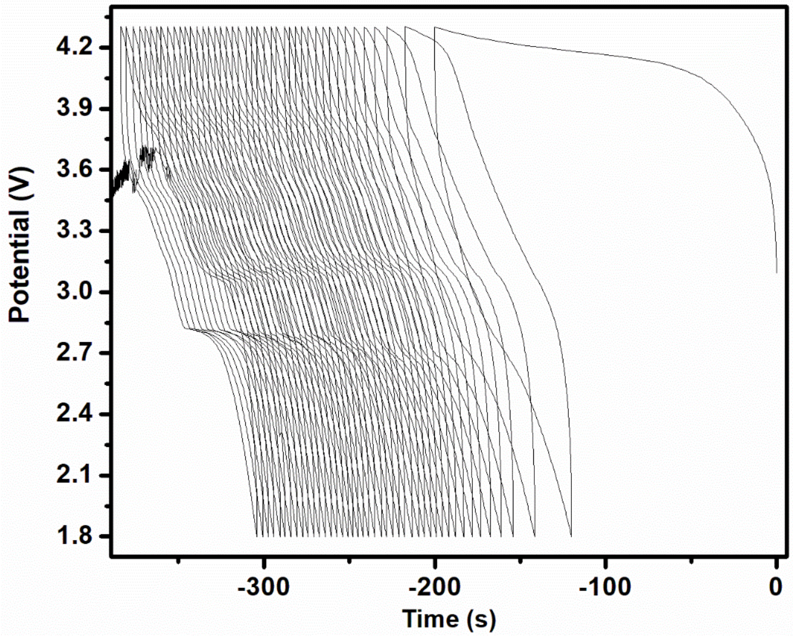

Figure S5. Potential vs. time plot of $Li_2Fe(C_2O_4)_2$ for the initial forty cycles.

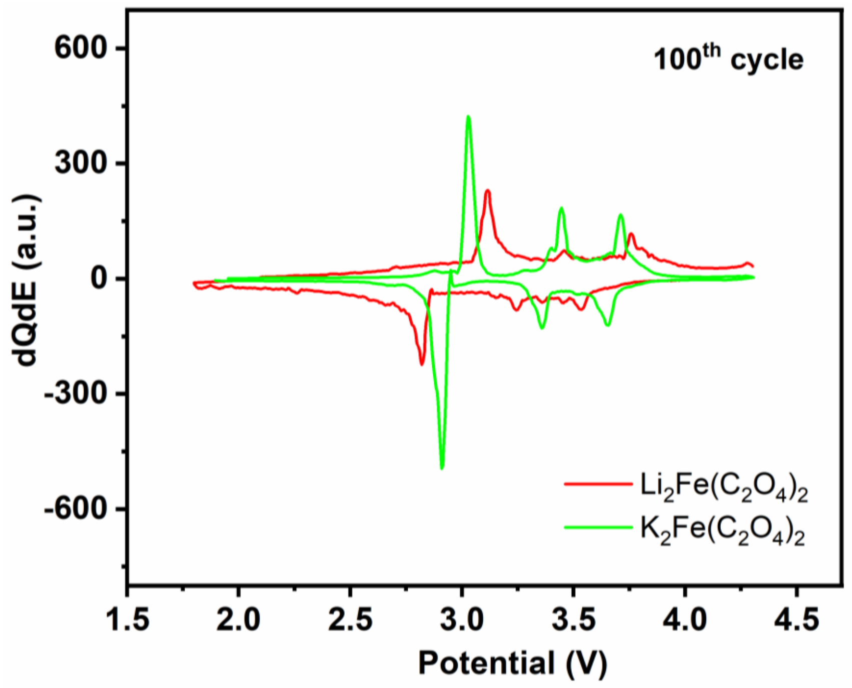

Figure S6. Differential capacity plots for the 100th cycle for both $Li_2Fe(C_2O_4)_2$ and $K_2Fe(C_2O_4)_2$.

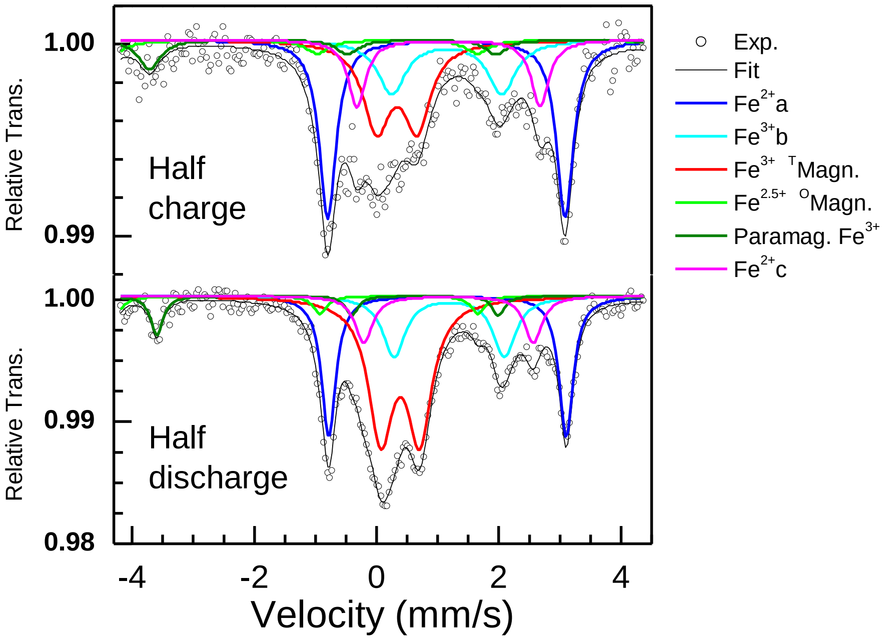

Figure S7. Half cycle (3.1 V) charge/discharge Mössbauer spectroscopy analysis for NIB.

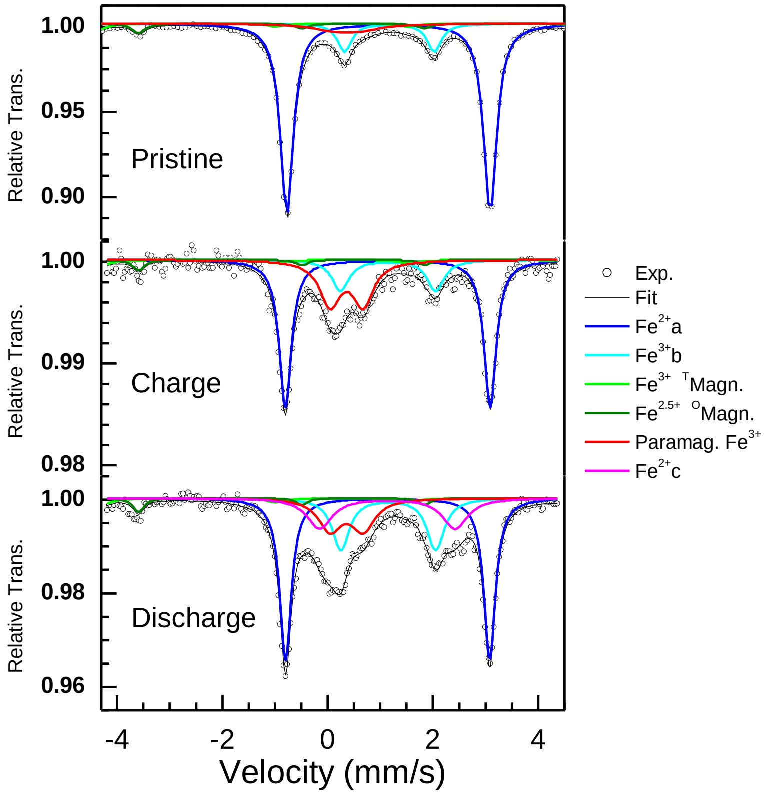

Figure S8. Mössbauer spectroscopy analysis for LIB at different states of charge.

Table S1: The isomer shift (IS), the quadrupole splitting (QS), the line width (LW), and the absorption (Abs) parameters from the fitting of the room temperature Mössbauer spectra obtained from pristine and carbon composite (Pristine/C65) samples.

Sample	Comp.	IS (mm/s)	QS (mm/s)	LW (mm/s)	H (Tesla)	Abs. (%)	Normalized abs. (%)
Pristine	Fe^{2+} a	1.16	3.87	0.30	-	64.6	79.4
	Fe^{2+} b	1.17	1.71	0.32	-	10.4	12.8
	Fe^{3+} para	0.37*	0.5*	1.00	-	6.4	7.9
	Magn. Tetra.	0.27	-	0.39	49.5	8.1	-
	Magn. Octa	0.68	-	0.32	45.8	10.5	-
Pristine /C65	Fe^{2+} a	1.17	3.87	0.29	-	57.6	76.0
	Fe^{2+} b	1.19	1.70	0.34	-	10.8	14.2
	Fe^{3+} para	0.37*	0.5*	0.69	-	7.4	9.8
	Magn. Tetra.	0.28	-	0.38	49.4	10.6	-
	Magn. Octa	0.68	-	0.34	45.8	13.6	-

Table S2: The isomer shift (IS), the quadrupole splitting (QS), the line width (LW), and the absorption (Abs) parameters from the fitting of the room temperature Mössbauer spectra obtained from full charge/discharge state samples.

Sample	Comp.	IS (mm/s)	QS (mm/s)	LW (mm/s)	Normalized Abs. (%)
Pristine	Fe^{2+} a	1.16	3.87	0.30	76.0
	Fe^{2+} b	1.17	1.71	0.32	14.2
	**Fe^{3+} para**	**0.37***	**0.5***	**1.00**	**9.8**
Charge	Fe^{2+} a	1.16	3.89	0.27	25.6
	Fe^{2+} b	1.17	1.80	0.41	16.7
	Fe^{2+} c	1.18	2.78	0.35	10.1
	**Fe^{3+} para**	**0.38**	**0.61**	**0.46**	**47.5**
Discharge	Fe^{2+} a	1.16	3.87	0.28	30.2
	Fe^{2+} b	1.17	2.03	0.62	20.0
	Fe^{2+} c	1.18	2.61	0.50	12.8
	**Fe^{3+} para**	**0.38**	**0.68**	**0.54**	**37.0**

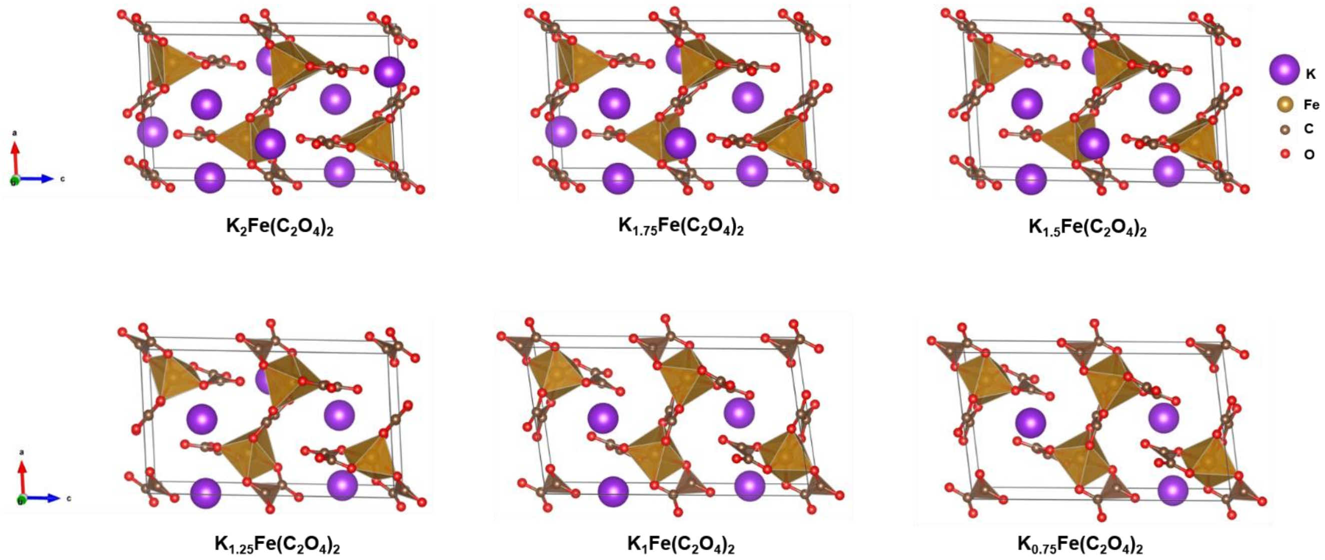

Figure S9. $K_xFe(C_2O_4)_2$ structure at different occupancies of K ions (x).

K-content (x) in $K_xFe(C_2O_4)_2$	a(Å)	b(Å)	c(Å)	β(°)
**2**	8.818	7.038	15.053	92.87
**1.75**	8.745	7.070	14.992	92.80
**1.5**	8.780	7.114	14.873	93.44
**1.25**	8.794	7.066	15.185	92.49
**1**	8.990	6.785	16.022	98.08
**0.75**	9.034	6.919	15.759	96.88

Table S3: Lattice parameters of $K_xFe(C_2O_4)_2$ structure at different occupancies of K ions (x) and vacancies.

Computational methodology to find the most stable configuration

Various structures can be generated for $K_{2-x}Fe(C_2O_4)_2$ based on the arrangements of K atoms. We identified the most stable atomic configuration for each structure, resulting in six variations of $K_{2-x}Fe(C_2O_4)_2$ (with x values of 0, 0.25, 0.5, 0.75, 1, and 1.25). This was accomplished by computing the electrostatic energy of all possible symmetrically unique atomic configurations using the Ewald summation method, then optimizing the geometry of the five structures with the lowest electrostatic energy using DFT and GGA+U, and finally selecting the most stable configurations by comparing the total energies of the structures previously calculated. The optimized structures' cell parameters are listed in Table S3.

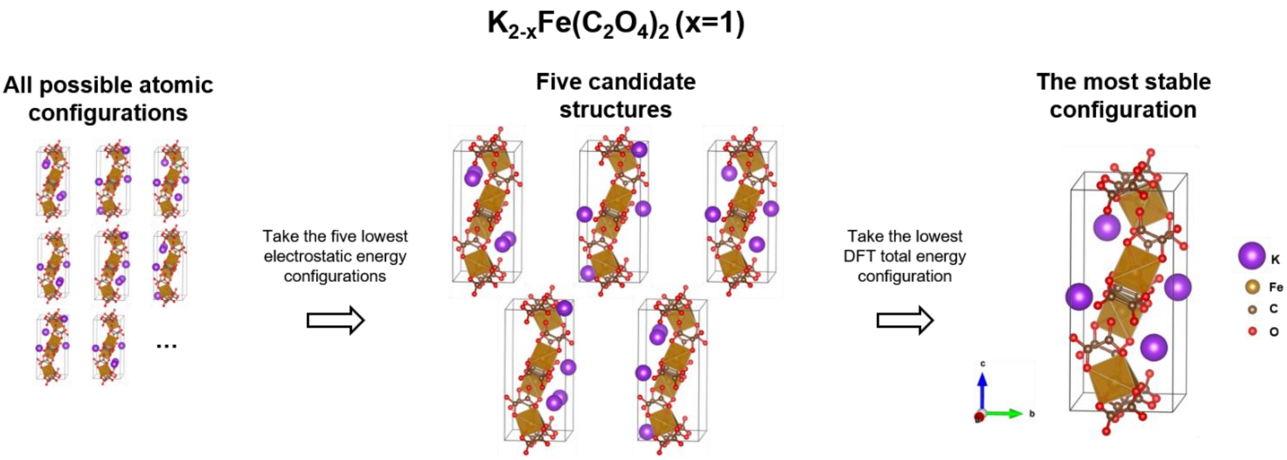

Figure S10. The modelling methodology of $K_{2-x}Fe(C_2O_4)_2$ (x=1) and the structures used in this study are presented schematically. The electrostatic energies of these structures were assessed using the Ewald summation method available in Pymatgen [DOI: 10.1016/j.commatsci.2012.10.028].

Configuration	1 (used for calculations)	2	3	4	5
Energy (eV)	-404.18	-405.49	-404.51	-404.49	-405.51

Table S4: Energy values of the different configurations chosen after electrostatic energy calculations.
